# Genomic Profiling on an Unselected Solid Tumor Population Reveals a Highly Mutated Wnt/β-Catenin Pathway Associated with Oncogenic EGFR Mutations

**DOI:** 10.3390/jpm8020013

**Published:** 2018-04-09

**Authors:** Jingrui Jiang, Alexei Protopopov, Ruobai Sun, Stephen Lyle, Meaghan Russell

**Affiliations:** 1 KEW Inc., 840 Memorial Drive, Cambridge, MA 02139, USA; rsun@kewinc.com (R.S.); slyle@kewinc.com (S.L.); mrussell@kewinc.com (M.R.); 2 Sanofi US, 270 Albany Street, Cambridge, MA 02139, USA; Alexei.Protopopov@sanofi.com

**Keywords:** NGS, EGFR-driven cancers, signaling, genomic profiling, biomarkers, Wnt/β-catenin pathway

## Abstract

Oncogenic epidermal growth factor receptors (EGFRs) can recruit key effectors in diverse cellular processes to propagate oncogenic signals. Targeted and combinational therapeutic strategies have been successfully applied for treating EGFR-driven cancers. However, a main challenge in EGFR therapies is drug resistance due to mutations, oncogenic shift, alternative signaling, and other potential mechanisms. To further understand the genetic alterations associated with oncogenic EGFRs and to provide further insight into optimal and personalized therapeutic strategies, we applied a proprietary comprehensive next-generation sequencing (NGS)-based assay of 435 genes to systematically study the genomic profiles of 1565 unselected solid cancer patient samples. We found that activating EGFR mutations were predominantly detected in lung cancer, particularly in non-small cell lung cancer (NSCLC). The mutational landscape of EGFR-driven tumors covered most key signaling pathways and biological processes. Strikingly, the Wnt/β-catenin pathway was highly mutated (48 variants detected in 46% of the EGFR-driven tumors), and its variant number topped that in the TP53/apoptosis and PI3K-AKT-mTOR pathways. Furthermore, an analysis of mutation distribution revealed a differential association pattern of gene mutations between *EGFR* exon 19del and EGFR L858R. Our results confirm the aggressive nature of the oncogenic EGFR-driven tumors and reassure that a combinational strategy should have advantages over an EGFR-targeted monotherapy and holds great promise for overcoming drug resistance.

## 1. Introduction

Epidermal growth factor receptor (EGFR) is a member of the HER/ERBB tyrosine kinase receptor family and plays critical roles in many biological processes and in tumorigenesis. Since the first discovery of its oncogenic kinase domain mutations [1,2], EGFR has been considered a key oncodriver for many tumor types, including non-small cell lung cancer (NSCLC) and colorectal cancer (CRC) [3]. Unlike its wild-type counterpart whose activation requires ligand binding, the clinically relevant oncogenic mutant EGFRs can constitutively, in the absence of ligand, activate themselves and the downstream pathways, such as the PI3K/AKT and MAPK pathways [4]. Furthermore, the constitutively activated mutant EGFRs are sensitive to their kinase inhibitors, such as gefitinib [4]. The targeted EGFR agents provide more treatment options and better therapeutic outcomes for the cancer patients whose tumors are driven by mutant EGFRs. However, the key clinical limitation of the anti-EGFR therapies is drug resistance [5].

The most common drug resistance mechanisms include mutations, oncogenic shift, alternative signaling, and histological changes. Src family of protein tyrosine kinases (SFKs) activity, aberrant expression of HER2, HER3, and cMET, oncogenic KRAS mutations, and other genetic alterations have all been shown to impact drug responses to anti-EGFR therapies [6,7,8,9,10,11,12]. Increased evidence shows that the Wnt/β-catenin pathway is abnormally activated in NSCLC and could be an important mechanism of drug resistance to EGFR inhibition. In NSCLC, β-catenin levels are increased in the cells harboring oncogenic EGFR mutations, as well as in gefitinib-resistant cells, and inhibition of the Wnt/β-catenin pathway re-sensitized cells to EGFR inhibitors and increased their efficacy [13,14,15].

Molecular profiling using next-generation sequencing (NGS) has been proven to be a powerful tool to discover driver events and to develop therapeutic strategies for optimal and personalized cancer treatments [16,17]. While developing next generation drugs is a promising strategy to overcome drug resistance, targeting other alternatives mechanisms that cancer cells become addicted to, such as oncogenic shift or alternative signaling pathways, is also an effective therapeutic option. To maximize the clinical benefit of targeted EGFR therapies, further understanding of the genetic alterations associated with activating EGFRs is important. In this study, we applied a proprietary NGS-based comprehensive genomic profiling assay [18] to systematically study the genomic profiles of an unselected solid cancer cohort of 1565 patients, with the purpose to uncover the distribution of activating EGFR mutations (including substitutions and small insertions and deletions) across tumor types, to further understand the genetic alterations associated with oncogenic EGFRs in an unbiased manner, and to provide further insight into optimal and personalized therapeutic strategies.

Our results show that activating EGFR mutations were predominantly detected in lung cancer, particularly in NSCLC, and co-occurred with the mutations of the genes participating in most key signaling pathways and biological processes, including receptors of different classes, key regulators involved in genome and epigenome stability, PI3K–AKT–mTOR pathway, and TP53/apoptosis pathway. Strikingly, the Wnt/β-catenin pathway-related genes were highly mutated in the context of oncogenic EGFRs, and the variant number of the Wnt/β-catenin pathway topped that in the TP53/apoptosis and PI3K–AKT–mTOR pathways. Our data also show that mutations in most of top three mutated genes were specifically enriched in the cases with actionable EGFR mutations: in particular, mutations in *RB1*, *JAK2*, *APC*, *JAK3*, *NF1*, and *SMAD4* were predominantly associated with activated mutant EGFRs. Interestingly, analysis of mutation distribution between EGFR L858R and *EGFR* exon 19del indicated that variants in several genes, including *ROS1*, *AXIN2*, *NLRP1*, *PIK3CA*, *TNK2*, *ABL1*, *RB1*, *PTCH1*, *ETV4*, *TFE3*, and phosphatases (*PTPRT* and *PTPRD*), were preferentially associated with *EGFR* exon 19del. The mutational signature of oncogenic EGFRs revealed in our study confirms the aggressive nature of the mutant EGFR-driven tumors. Together, our results underscore the clinical advantage of combinational therapy over EGFR-targeted monotherapy and point to the great promise of combining targeted EGFR therapy with modulators targeting several pathways, including the Wnt/β-catenin, TP53/apoptosis, and PI3K–AKT–mTOR pathways, to overcome drug resistance.

## 2. Results and Discussions

### 2.1. Classification of a Mutant EGFR Containing Cancer Cohort

**Figure 1:**
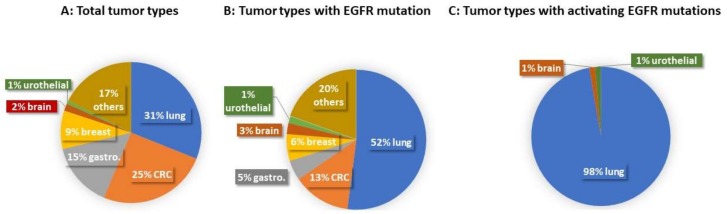
Tumor Types and Distribution of EGFR Mutations. Figure 1 is based on Table 1. Figure 1A shows the percentages of tumor types in our cohort: 31% lung cancer, 25% CRC, 15% gastroesophageal adenocarcinoma, 9% breast cancer, 2% brain tumor, 1% urothelial carcinoma, and 17% other solid tumors; Figure 1B shows the percentages of tumor types associated with EGFR mutations: 52% lung cancer, 13% CRC, 6% breast cancer, 5% gastroesophageal adenocarcinoma, 3% brain tumor, 1% urothelial carcinoma, and 20% other solid tumor types; Figure 1C shows the percentages of tumor types associated with activating EGFR mutations: 98% lung cancer, 1% brain tumor, and 1% urothelial carcinoma.

CANCERPLEX [18] was applied to analyze 1565 cancer samples from an unrestricted solid tumor patient population. A total of 194 samples harbored EGFR mutations (194/1565) (Table 1 and Figure 1), predominantly found in lung cancer (101/194). The 194 mutant EGFR-harboring samples were further classified into two categories based on the functional and pathological impacts of the mutations. The cases in the first category harbored mutant EGFRs that were not oncogenic, had not been reported previously, or had unknown significance. The cases in the second category harbored actionable mutant EGFRs (AE group), meaning that these mutations have been demonstrated to be oncogenic and/or respond to EGFR-targeted therapies. A total of 74 cases (74/194) harbored actionable EGFR mutations and belonged to the AE group. Among the 74 actionable mutant EGFR-containing tumors, there were 72 cases of lung cancer (69 NSCLC plus 3 lung cancers (historical)) 1 brain tumor (glioblastoma), and 1 urothelial carcinoma (Table 1 and Figure 1). The activating EGFR mutation types in our cohort were predominantly exon 19 deletions (40/74) and exon 21 L858R missense point mutation (25/74) (Table 2), which are two of the most commonly found mutations in lung cancer [19]. While the majority of cases in the AE group harbored a single activating EGFR mutation, four NSCLC adenocarcinoma cases harbored double activating EGFR mutations: exon 19 p.746_750del + exon 20 p.T790M (two cases), exon 21 p.L858R + exon 18 p.E709K (one case), and exon 18 p.G719A + exon 20 p.R776H (one case). Three out of four double activating EGFR mutations containing cases are the Asian population.

### 2.2. Functional Analysis and Mapping of the Variants in the AE Group

The variants in the AE groups were then subjected to a functional analysis and mapping based on the known primary function of the genes in the signaling pathways or biological processes (Table 3). Our results show that the mutational landscape of EGFR-driven tumors covered key signaling pathways and biological processes. In the context of activating EGFR mutations, the top mutated genes were those encoding receptors and effectors involved in genome and epigenome stability. As expected, the variants were also found in the genes involved in common pathways and biological processes, such as proliferation, apoptosis, and cell cycle progression. Of most interest, we found that the Wnt/β-catenin pathway-related genes were mutated to a greater extent, as compared with the known downstream targets/pathways of EGFR, such as the PI3K–AKT and the TP53/apoptosis pathways.

#### 2.2.1. Receptors

Receptors of different classes were clustered together. The majority of the variants were found in genes encoding surface receptors. A total of 189 variants were detected in 93% (69/74) of the tumors in the AE group (AE tumors). The top three mutated receptor genes were *PKHD1* (21 variants), *ROS1* (18 variants), and *RET* (9 variants) (Table 3 and Table 4) and their mutation types are listed in Table 4.

**PKHD1** and EGFR are linked in autosomal recessive polycystic kidney disease (ARPKD), a disease caused by PKHD1 mutations and in which EGFR signaling is overactivated. Silencing PKHD1 in ARPKD models caused abnormal proliferation due to hyperactivation of the EGF-induced ERK1/2 [20]. PKHD1 has been implicated in CRC tumorigenesis because of the high level of *PKHD1* mutations [21]. It is well known that EGFR is overexpressed to a great extent in CRC, and anti-EGFR drugs are a golden standard therapy for RAS wildtype metastatic CRC [3,22]. It will be interesting to determine whether the high level of *PKHD1* mutations found in CRC is due to EGFR overactivity.

**ROS1** is well known because of its fusions with diverse partners that result in its constitutive kinase activation. In general, *ROS1* fusion is exclusive to other driver mutations, such as *EGFR*, *ALK*, and *KRAS*. However, the concurrence of an activating *EGFR* mutant and oncogenic *ROS1* fusions has been reported in lung cancer and results in reducing the therapeutic efficacy of EGFR tyrosine kinase inhibitors (TKIs) used as a monotherapy [23,24,25]. Our finding that *ROS1* mutations co-exist with activating *EGFR* mutations to a great extent supports the notion that co-inhibiting ROS1 and EGFR could be a promising alternative for the treatment of NSCLC patients with concurrent mutations of these two genes.

**RET** and EGFR interaction has been identified in a subset of NSCLC adenocarcinomas, in which EGFR is a key regulator of RET activation; also, EGF could trigger resistance to RET inhibition in CCDC6-RET lung cancer cells [26,27]. An in-frame fusion of CCDC6-RET was detected in two EGFR-mutated NSCLC tumors progressing after EGFR-TKI treatment, but not in the tumors before the EGFR-TKI treatment. It was suggested that such EGFR-TKI treatment-induced *RET* rearrangement is a potential mechanism of acquired drug resistance to EGFR-TKI [28].

#### 2.2.2. Genes Involved in Genomic and Epigenomic Stability

Given the critical role of genome and epigenome instability in oncogenesis and anti-cancer drug response, genes involved in these biological processes, such as DNA damage response (DNA mismatch repair (MMR), nucleotide excision repair (NER)), chromatin remodeling, methylation and acetylation, etc., were clustered into a group. The genes in this group were also highly mutated in the context of activating EGFR mutations. A total of 182 variants were detected in 88% (65/74) of the AE tumors. The top three mutated genes are *ARID1A* (17 variants), *ATM* (13 variants), and *BRCA2* (11 variants) (Table 3 and Table 4), and their mutation types are listed in Table 4.

**ARID1A** acts as a gatekeeper for genome stability whose loss is linked to malignant transformation and is a statistically significant mutated gene in lung adenocarcinoma [29,30]. ARID1A is also one of the three SWI/SNF subunits that contributes to EGFR dependence in NSCLC, whose loss might confer the host cells resistance to EGFR inhibitors [31]. Our results further underscore the importance of ARID1A in lung tumorigenesis, particularly in EGFR-driven lung cancer.

**ATM** is a central player in the repair of DNA double-strand breaks (DSB) and, therefore, in genome stability. *ATM* gene mutations are commonly found in lung adenocarcinoma and are associated with increased cancer risk [32,33]. EGFR can bind to and activate ATM in response to DNA damage, which in turn initiates genome surveillance programs, and EGFR inhibition abolishes such response and increases tumor radiosensitivity [34]. Our finding that *ATM* is the second commonly mutated gene among the genes involved in genome surveillance further suggests the direct involvement of mutant EGFRs in genome instability.

**BRCA2** is a tumor suppressor that participates in genome surveillance programs. Germline mutations in the *BRCA* genes are strongly associated with an elevated risk of breast and ovarian cancers. While the role of BRCA in lung cancer has not been studied systematically, there are several reports of lung cancer cases with the concurrence of germline BRCA2 frameshift mutations and activating EGFR mutations, suggesting that mutant BRCA2 could be a promising genome marker for prognosis, diagnosis of lung cancer, and combinational treatment options with EGFR inhibition [35,36]. Our finding that *BRCA2* mutations often co-exist with activating mutant EGFRs, suggests a potential role of BRCA2 in EGFR-driven tumorigenesis.

#### 2.2.3. Wnt/Β-Catenin Pathway

Strikingly, our results show that the genes involved in the Wnt/β-catenin pathway were mutated to a greater extent, as compared with those in the TP53/apoptosis and PI3K–AKT–mTOR pathways. The fundamental role of the Wnt/β-catenin pathway in development requires its tightly control at multiple levels, and thus a defect at any level may contribute to tumorigenesis. The Wnt/β-catenin pathway is emerging as an important player in NSCLC, also because of its involvement in conferring resistance to therapies [14,37,38]. Growing evidence shows that the crosstalk between the Wnt and EGFR pathways contributes to lung tumorigenesis and drug resistance [39]. In EGFR-driven NSCLC, tumors with unmethylated Wnt antagonist genes are significantly associated with a good prognosis, as compared to those tumors with methylated, hence inactivated, Wnt antagonist genes [40]. It has been demonstrated that mutant EGFR and β-catenin can interact directly leading to increased nuclear localization of β-catenin and of its transcriptional activity [41]. A total of 48 variants were detected in 46% (34/74) of the AE tumors. The top three mutated genes involved in the Wnt/β-catenin pathways were *FAT1* (12 variants), *APC* and *RNF43* (7 variants respectively), and *AXIN2* (6 variants) (Table 3 and Table 4), and their mutation types are listed in Table 4.

**FAT1** is a tumor suppressor and plays an important role in Wnt/β-catenin signaling by binding directly to β-catenin to prevent its nuclear localization [42,43,44]. Although *FAT1* expression is upregulated in acute myeloid leukemia (AML) and acute lymphoblastic leukemia (ALL) [45], emerging evidence has shown that FAT1 loss induces aberrant activation of the Wnt/β-catenin pathway and promotes tumorigenesis, particularly in solid tumors [43,44,46]. The fact that both FAT1 and mutant EGFR can bind to β-catenin directly suggest that mutant EGFR might compete with FAT1 for β-catenin binding, thus impacting on the aberrant activation of the Wnt/β-catenin pathway.

**RNF43** negatively regulates the Wnt/β-catenin pathway by direct interaction with either the upstream Wnt receptors of the Frizzled family or the downstream effector TCF4, a transcriptional co-activator of β-catenin. Binding of RNF43 to TCF4 silences TCF4 transcriptional activity even in the presence of constitutively active mutants of β-catenin [47]. Deleterious *RNF43* mutations are implicated as drivers of Wnt-dependent tumor growth and are mutually exclusive to *APC* mutations, especially in CRC [48,49].

**APC** is a negative regulator of Wnt signaling and its loss is responsible for most sporadic and hereditary forms of CRC due to aberrant activation of the Wnt/β-catenin pathway [50]. The connection between EGFR and APC is mainly found in CRC. In APC-null CRC mice model, EGFR kinase activity and PI3K–AKT signaling are increased. APC deficiency is associated with increased EGFR activity in intestinal enterocytes and adenomas of C57BL/6J-Min/+ mice [51]. Our results suggest that APC may coordinate with mutant EGFR signaling to affect lung tumorigenesis.

**AXIN2** acts as a negative regulator of the Wnt/β-catenin pathway under physiological conditions [52]. However, its tumor suppressive and oncogenic activities seem to be context-dependent and tumor type-specific [53,54,55]. AXIN2 is both a component of cellular β-catenin destruction box and a β-catenin target gene and, therefore, participates in a negative feedback loop to limit Wnt-initiated signalling [52,56]. In vitro, EGFR inhibition downregulated the expression of *AXIN2* [57]. It is possible that oncogenic mutant EGFRs can also impact on the aberrant activation of the Wnt/β-catenin pathway by interfering with such negative feedback loop.

#### 2.2.4. TP53/Apoptosis and RB/Cell Cycle Pathways

The TP53 and Rb are two prototypical tumor suppressors that control two key complementary cellular regulatory networks to maintain tissue homeostasis and to decide cell fate, and dysregulations of these two pathways are hallmarks of cancers [58,59]. As expected, mutations were also found in the TP53/apoptosis and Rb/cell cycle pathways; however, the variant numbers were significantly different. A total of 40 variants in the TP53/apoptosis axis were detected in 45% (33/74) of the AE tumors, whereas 19 variants in the Rb/cell cycle axis were detected in only 24% (18/74) of the AE tumors. The top three mutated genes in the TP53/apoptosis axis were *TP53* (27 variants), *NUMA1* (5 variants), and *NLRP1* (3 variants), and the top three mutated genes in the Rb/cell cycle axis were *Rb* and *NUP214* (4 variants, respectively), *CDKN2A* and *CDK12* (2 variants, respectively), and *CCND1*, *2*, *3*, etc., each with only one variant (Table 3 and Table 4); their mutation types are listed in Table 4.

The interaction between EGFR and the TP53/apoptosis pathway has long been recognized. It has been shown that some cancer cells that naturally express high levels of EGFR preferentially undergo apoptosis upon EGF ligand activation [60], and suppressing EGFR signaling may prime neoplastic cells for apoptosis induced by other cytotoxic stimuli, such as chemotherapy drugs and radiation [61]. On the other hand, in normal human keratinocytes, p53 loss induces EGFR expression [62], and mutant p53 amplifies EGFR family signaling to promote mammary tumorigenesis [63]. In NSCLC cells, p53 loss is associated with drug resistance to EGFR inhibitors and radiation [64].

While the Rb/cell cycle pathway has a crucial role in lung tumorigenesis and is impaired in almost all lung cancers [65], the most notable interaction between the Rb/cell cycle pathway and EGFR is represented by the role of Rb in the small cell lung cancer transformation. The transformation of EGFR mutant lung adenocarcinoma (LADC) into small cell lung cancer (SCLC) constitutes one of the major resistant mechanisms to EGFR-TKI. Rb loss is necessary for such transformation but not sufficient to promote the process [66], and complete inactivation of both RB1 and TP53 in LADC is the prerequisite [67].

Our finding confirms the universal role of the p53/apoptosis pathway in transducing oncogenic signals of the mutant EGFRs and warrants further study of this pathway in mutant EGFR-driven tumors for a rational development of combinational treatment strategies involving p53 restoration, EGFR inhibition, and radiation.

#### 2.2.5. PI3K–AKT–mTOR and RAS–RAF–MEK–ERK Pathways

The PI3K–AKT–mTOR and RAS–RAF–MEK–ERK pathways represent the chief mechanisms for cells to regulate survival, proliferation, and motility in response to extracellular signals, in an independent yet interrelated manner [68]. As the key players in controlling cell fate, these two pathways are often aberrantly activated during oncogenesis [4,69]. As expected, mutations of genes involved in these two pathways were also found in a subset of EGFR-mutated tumors, but the variant numbers were significantly different. A total of 40 variants in the PI3K–AKT–mTOR pathway were detected in 42% (31/74) of the AE tumors, whereas 19 variants in the RAS–RAF–MEK–ERK pathway were detected in only 24% (18/74) of the AE tumors. The top three mutated genes in the PI3K–AKT–mTOR pathway were *PIK3CA* (10 variants), *RICTOR* (5 variants), and *PIK3R2* and *TSC2* (3 variants, respectively), and the top three mutated genes in the RAS–RAF–MEK–ERK pathway were *NF1* and *RASA1* (5 variants, respectively), *KIAA1804* (3 variants), and *RPS6KA2* (2 variants) (Table 3 and Table 4); their mutation types are listed in Table 4.

It has been shown that EGFR-mediated mitogenic signaling appears to depend on the activation of both JAK-2 and PI3K pathways, but EGFR kinase activity seems not required for initiating the activation of the RAS–ERK pathway [70]. Also the constitutively dimerized mutant EGFRvIII preferentially activates the PI3K pathway over other pathways such as the MAPK pathway [69]. In prostate cancer cells, AKT signaling has a pivotal role over ERK signaling in EGFR-mediated cell migration by activating epithelial–mesenchymal transition (EMT) [71]. In mutant EGFR-driven NSCLC, MEK-induced apoptosis is dependent on the inhibition of the PI3K pathway to downregulate Mcl-1 protein, a major survival protein, which then sensitizes the cells to MEK inhibition [72]. In EGFR-mutated LADC cells, PI3K–AKT–mTOR signaling is indispensable for the regulation of aerobic glycolysis, whereas RAS–MEK–ERK signaling has a limited effect in the process [73]. These studies suggest that, although mutant EGFR can activate both the PI3K and MAPK pathways, the PI3K signaling seems to play a pivotal role in transducing mutant EGFR oncogenic signaling, which might explain our finding that more variants were detected in the PI3K–AKT–mTOR pathway than in the RAS–RAF–MEK–ERK pathway. This finding also supports the notion that, after the p53 pathway, the PI3K–AKT–mTOR pathway is one of the most mutated pathways associated with human cancer [74,75].

#### 2.2.6. Kinase and Phosphatase

Other kinases and phosphatases that have not been assigned in this study belong to this group. These two groups of enzymes catalyze the opposite reactions of protein phosphorylation and dephosphorylation, which are the most prevalent posttranslational modifications that control a wide range of cellular processes by regulating the structure and function of cellular proteins [74,76]. Many more variants in the kinase group were found as compared with the phosphatase group. A total of 32 variants in the kinase group were detected in 34% (25/74) of the AE tumors, whereas only 5 variants in the phosphatase group were detected in only 12% (4/74) of the AE tumors. The top three mutated kinase genes were *TNK2* and *TYK2* (8 variants, respectively), *ABL1* and *JAK3* (4 variants, respectively), and *JAK2* (3 variants) (Table 3 and Table 4), and their mutation types are listed in Table 4. Only two phosphatase genes were found mutated, namely, *PTPRT* with four variants and *PTPRD* with only one variant (Table 3 and Table 4); their mutation types are listed in Table 4.

Even though many different kinase genes and significantly fewer phosphatase genes are found in an organism under physiological conditions, the activities of these kinases are well balanced by a small set of phosphatases who seem to balance their reduced gene numbers through high protein abundance [77,78]. Our finding of much higher variant numbers in the kinase genes might reflect the nature of the unbalanced numbers of kinase genes and phosphatase genes, yet it might also suggest that activated mutant EGFRs preferentially induce mutations of certain kinases to offset the balance of kinases and phosphatases and propagate the oncogenic signals.

#### 2.2.7. NOTCH

A total of 34 variants in the NOTCH pathway were detected in 35% (26/74) of the AE tumors. The top three mutated genes were *NOTCH2* and *NOTCH4* (8 variants, respectively), *SPEN* (7 variants), and *MAML2* (6 variants) (Table 3 and Table 4), and their mutation types are listed in Table 4. EGFR and NOTCH pathways are both important in cell proliferation, differentiation, and apoptosis, and have a fundamental role during the development of multicellular organisms. The interaction of these two pathways has been reported in diverse biological processes with significant phenotypic outcomes, in particular, drug resistance [79,80,81,82]. Our finding that the genes of the NOTCH pathways were mutated to a substantial degree in the context of activated mutant EGFRs further confirm the notion that a combinational treatment consisting of EGFR and NOTCH inhibition could be a promising therapeutic strategy for NSCLC patients.

#### 2.2.8. Others (Hedgehog, NF-κB, TGF-β)

Although to a lesser extent, variants of the genes in the pathways of Hedgehog (Hh) (12 variants detected in 14% (11/74) of the AE tumors), NF-κB (10 variants detected in 12% (9/74) of the AE tumors), and TGF-β (9 variants detected in 11% (8/74) of the AE tumors) were also detected in our cohort (Table 3 and Table 4).

The Hh pathway participates in organ development and stem cell maintenance [83]. One of the most notable results of the interaction between hedgehog (Hh) and EGFR signaling is in the resistance to the EGFR inhibition via EMT induction, and targeting Hh pathway may lead to the reversal of the EMT phenotype and improve the therapeutic efficacy of EGFR-TKIs in NSCLC [84,85,86,87].

NF-κB signaling plays a key role in immune and inflammation responses and its dysregulation contributes to several diseases, including cancer [88].The NF-κB pathway can be directly activated by EGFR and synergizes with EGFR signaling to promote lung tumorigenesis [89,90], particularly involving the resistance to EGFR inhibition via different mechanisms [91,92,93].

TGF-β signaling involves many cellular processes and plays an important role in EMT [94]. The most significant impact of TGF-β signaling in mutant EGFR signaling is its role in the resistance to EGFR inhibition through the induction of EMT. Cancer cells can counteract EGFR inhibition by increasing TGF-β production to induce EMT and EGFR-TKI resistance [94,95,96].

Given that the crosstalk between EGFR and the Hh, NF-κB, or TGF-β pathways may impact on the resistance to EGFR inhibition, it would be interesting to determine whether the number of variants in these three pathways increases in EGFR-TKI-treated or -resistant tumors.

### 2.3. The Mutation Types of the Top Three Mutated Genes and Their Enrichment in AE Cases

We next looked at the mutation types of the top three mutated genes associated with activated mutant EGFRs in each category and the fold change of variant frequency in 74 AE cases compared to the frequency in the total 1565 cases, as showed in Table 4. Except for *PIK3R2*, *ABL1*, *CCND3*, and *REL*, whose fold change of frequency (FC) in the AE cases were between 0.7 and 1, as well as *BRCA2* (FC = 1.2), *ARID1A* (FC = 1.7), and *RET* (FC = 1.8), all other top three mutated genes (88%) showed a more than 2-fold higher total mutation frequency in the AE cases, indicating that these mutations were specifically enriched in the AE cases of our study. In particular, more than 60% of the mutations in the following genes were exclusively detected in the AE cases: *RB1* (100%, 4/4, p.L171fs, p.F482fs, p.K192E, p.A538fs), *JAK2* (100%, 3/3, p.Y1099C, p.S797C, p.Q955R), *APC* (70%, 7/10), *JAK3* (67%, 4/6), *NF1* (83%, 5/6), and *SMAD4* (67%, 2/3). The mutations of several genes with fewer than five detected mutations, including a single mutation of *AURKA* (p.G173R), *SUFU* (p.R239W), *TNFAIP3* (p.C590S), *BCL11B* (p.D122N), *PTPRD* (p.R1088H1), and two mutations of *ACVR2A* (p.M148T and p.A151V), were exclusively found in the AE cases. In addition, several mutations in most of the genes with more than five mutations detected were also exclusively associated with activated mutant EGFRs, as shown in Table 4.

Further analysis of those seven genes with FC below 2 indicated that the low FC was due to one specific mutation detected with high frequency in total cases but with low frequency in the AE cases. In most instances, these mutations were benign (*BRCA2* M784V and *RET* D489N), germline polymorphisms (*PIK3R2* P4S and *CCND3* E253D), sequencing artifacts (*ARID1A* L117P), or likely had minimum functional impacts (*ABL1* K609del and *REL* L331S).

### 2.4. Mutation Distribution of Top Three Mutated Genes between EGFR L858R and EGFR Exon 19del

EGFR L858R and *EGFR* exon 19del are two of the most commonly found EGFR oncogenic mutations, particularly in lung cancer [19] and were also predominantly detected in our cohort (Table 2). To explore further whether there were particular patterns of association between the top three mutated genes and these two specific EGFR mutations, we next examined how the mutations of the top three mutated genes were distributed in EGFR L858R and *EGFR* exon 19del tumors, as shown in Table 5. Our analysis showed that most mutations of the top three mutated genes in the groups of Genome and Epigenome, NOTCH, and RAS-RAF-MEK-ERK did not show a preferential association with either EGFR L858R or *EGFR* exon 19del. Also, EGFR L858R mutation did not show a preferential association with any of the most specific gene alterations, except for single mutations of several genes, including *CHEK1* (p.V46I), *BUB1B* (p.R550Q), IKBKE (two mutations, p.V418M and p.T483M), *CARD11* (p.M551T), *BCL11B* (p.D122N), *BLNK* (p.I308T), and *SMAD4* (p.M447fs), that were exclusively associated with EGFR L858R. However, our results did show a specific mutational pattern associated with *EGFR* exon 19del. The mutations of the genes predominantly associated with *EGFR* exon 19del included *ROS1* (fold changes of variant frequency: exon 19del:L858R =1.3:0.5), *AXIN2* (1:0), *NLRP1* (1.2:0), *PIK3CA* (1.5:0.3), *TNK2* (1.2:0), *ABL1* (1.4:0), *RB1* (1.4:0.7), *PTCH1* (1.85:0), *ETV4* (1.2:0), *TFE3* (1.85:0), and *PTPRT* (1.4:0.7). In addition, several single mutations found in *CCND1* (p.276_276del), *CCND2* (p.R262H), *CCND3* (p.E253D), *AURKA* (p.G173R), *CDKN1A* (p.P4L), *STK36* (p.H713Y), *SUFU* (p.R239W), *IKBKB* (p.M83V), *REL* (p.L331S), *TNFAIP3* (p.C590S), *BIRC3* (p.R202S), and *PTPRD* (p.R1088H1) were also exclusively associated with *EGFR* exon 19del.

In this study, we clustered concurrent variants with activating mutant EGFRs on the basis of the known primary functions of the examined genes in signaling pathways and/or biological processes. We found that receptors of different classes and effectors involved in genome and epigenome stability were mutated to the highest degree in the context of activating EGFR mutations, which suggests that mutant EGFRs may augment their oncogenic signals by disrupting genome and epigenome stability and recruiting more receptors into the neoplastic transformation processes. The analysis of the fold change of variant frequency in 74 AE cases as compared to the frequency in the total 1565 cases showed a specific enrichment of the majority of mutations in the AE cases, particularly, the mutations *of RB1*, *JAK2*, *APC*, *JAK3*, *NF1*, and *SMAD4*. The analysis of mutation distribution revealed that *EGFR* exon 19del, as compared with EGFR L858R, was preferentially associated with mutations of *ROS1*, *AXIN2*, *NLRP1*, *PIK3CA*, *TNK2*, *ABL1*, *RB1*, *PTCH1*, *ETV4*, *TFE3*, and phosphatases (*PTPRT* and *PTPRD*). Our results also suggest that activated mutant EGFRs appear preferentially to associate with the TP53/apoptosis over Rb/cell cycle pathways and the PI3K–AKT–mTOR pathway over the RAS–RAF–MEK–ERK pathway. Unexpectedly, we found that the Wnt/β-catenin pathway was mutated to a high degree, even higher than that of the TP53/apoptosis and PI3K–AKT–mTOR pathways. Increased evidence has shown that the Wnt/β-catenin and oncogenic EGFR signaling cascades interact with each other in lung tumorigenesis and drug resistance [38,39,41]. Our results suggest that more in-depth studies of the Wnt/β-catenin pathway in mutant EGFR-driven tumors are needed in order to understand the tumorigenic impact and the mechanisms of drug resistance of the crosstalk between these two pathways and to develop combinational treatment strategies implying the co-inhibition of oncogenic mutant EGFRs and the aberrantly activated Wnt/β-catenin pathway.

## 3. Materials and Methods

### 3.1. Patient Samples

A total of 1565 cancer patient samples were obtained from cancer centers and hospitals in the US, Japan, Israel, and China. The samples were from an unselected patient population, in terms of solid tumor types and stages, age, sex, and ethnicity. This study was conducted on de-identified data under Institutional Review Board (IRB) protocol number: KEW-CR-001, and the NGS-based analysis was performed in a CLIA/CAP-accredited laboratory (KEW Inc., Cambridge, MA, USA).

### 3.2. CANCERPLEX

A proprietary NGS assay system, CANCERPLEX, was applied for the analysis of the patient samples [18]. Briefly, the formalin-fixed paraffin-embedded (FFPE) specimen blocks were dissected, and the tissue sections were reviewed by a pathologist for tumor purity; high purity tumor sections (20% of tumor purity as the threshold) were selected for genomic DNA extraction. An NGS assay with a targeted, full-gene sequencing of 435 cancer genes for detection of single nucleotide variants (SNVs), indels, structural abnormalities, microsatellite instability (MSI) markers, and estimation of the mutation burden, was then applied for sequencing using Illumina MiSeq and NextSeq platforms with average 500× sequencing depth. The raw sequencing data were processed through a bioinformatics pipeline (GENEPIPER), and the qualified calls/data were uploaded to a knowledgebase (GENEKEEPER) before functional analysis.

### 3.3. Functional Mapping of the Variants to Their Respective Pathways

Each gene with qualified variants from the resulting data set was then subjected to functional mapping and assigned to a signaling pathway or a biological process on the basis of the gene’s primary function. Several databases were used for functional mapping, including but not limited to: COSMIC, UniProt, KEGG, OMIM, OncoKB, JAX-CKB, PMKB, PubMed, and NCBI ClinVar.

## Figures and Tables

**Table 1:** Tumor Types and Distribution of Epidermal Growth Factor Receptor (EGFR) Mutations.

Case Type	Total Case (Number)	Lung Cancer	CRC	Gastroesophageal Adenocarcinoma	Breast Cancer	Brain Tumor	Urothelial Carcinoma	Others
Total tumor types	1565	486	395	234	138	26	12	274
Tumor types with EGFR mutations	194	101	26	9	12	5	3	38
Tumor types with activating EGFR mutations	74	72				1	1	

CRC: colorectal cancer; A total of 1565 cancer patient samples from an unselected patient population, in terms of solid tumor types and stages, age, sex, and ethnicity were subjected to an analysis using a proprietary comprehensive NGS-based assay; Table 1 shows: (1) The tumor types in the 1565 cases; (2) The tumor types in the 194 (194/1565) cases associated with EGFR mutations; (3) The tumor types in the 74 (74/194) cases associated with activating EGFR mutations, including 72 (72/74) cases of lung cancers, 1 (1/74) case of brain tumor (glioblastoma), and 1 (1/74) case of urothelial carcinoma.

**Table 2:** Activating EGFR Mutation Types and Associated Tumor Types.

EGFR Mutation Types	Case Number	Tumor Types (Case Number)
EGFR exon 21 p.L858R	24	NSCLC (8 adeno + 15 NOS) + 1 Lung cancer (Historical)
EGFR exon 21 p.L858R + exon 18 p.E709K	1	NSCLC (1 adeno)
EGFR Exon 19 p.746_750del	25	NSCLC (10 adeno + 3 sequam + 10 NOS) + 2 Lung cancer (Hisorical)
EGFR Exon 19 p.746_750del + Exon 20 p.T790M	2	NSCLC (2 adeno)
EGFR Exon 19 p.746_751del	4	NSCLC (3 adeno + 1 NOS)
EGFR exon 19 p.746_752del	1	NSCLC (1 adeno)
EGFR exon 19 p.746_748del	2	NSCLC (2 adeno)
EGFR exon 19 p.L747_T751del	1	NSCLC (1 adeno)
EGFR exon 19 p.747_752del	1	NSCLC (1 adeno)
EGFR Exon 19 p.747_753del	4	NSCLC (3 adeno + 1NOS)
EGFR exon 17 p.S645C	1	1 Brain tumor: Glioblastoma
EGFR Exon 18 p.G719A	1	NSCLC (1 adeno)
EGFR Exon 18 p.G719A + exon 20 p.R776H	1	NSCLC (1 adeno)
EGFR Exon 20 p.A767delinsASVD	3	NSCLC (1 adeno + 1 sequam) + 1 Urothelial carcinoma
EGFR exon 20 p.N771delinsNPHVC	1	NSCLC (1 adeno)
EGFR exon 20 p.M766delinsMASV	1	NSCLC (1 adeno)
EGFR exon 21 p.L861Q	1	NSCLC (1 adeno)
Total Case	74	

NSCLC: non-small cell lung cancer; The activating EGFR mutation types detected in 74 cases were exon 21 p.L858R in 25 cases (including one double mutation); exon 19 deletions in 40 cases (including two double mutations); other activating mutations located in exon 17, exon 18, exon 20, and exon 2 in nine cases.

**Table 3:** Mutated Pathways Associated with Activated Mutant EGFRs.

Associated Case Number	Pathway/Biological Function	Total Variant Number	TOP 1 Mutated Gene (Variant Number)	TOP 2 Mutated Gene (Variant Number)	TOP 3 Mutated Gene (Variant Number)
69	Receptors	189	*PKHD1* (21)	*ROS1* (18)	*RET* (9)
65	Genome and Epigenome	182	*ARID1A* (17)	*ATM* (13)	*BRCA2* (11)
34	Wnt/β-catenin	48	*FAT1* (12)	*APC*, *RNF43*, (7)	*AXIN2* (6)
33	TP53/apoptosis	40	*TP53* (27)	*NUMA1* (5)	*NLRP1* (3)
31	PI3K-AKT-mTOR	40	*PIK3CA* (10)	*RICTOR* (5)	*PIK3R2*, *TSC2*, (3)
26	NOTCH	34	*NOTCH2*, *NOTCH4*, (8)	*SPEN* (7)	*MAML2* (6)
25	Kinase	32	*TNK2*, *TYK2*, (8)	*ABL1*, *JAK3*, (4)	*JAK2* (3)
18	Rb1/cell cycle	19	*Rb*, *NUP214*, (4)	*CDKN2A*, *CDK12*, (2)	*CCND1*, *2*, *3*, et al., (1)
18	RAS-RAF-MEK-ERK	19	*NF1*, *RASA1*, (5)	*KIAA1804* (3)	*RPS6KA2* (2)
11	Hedgehog	12	*PTCH1* (4)	*SMO*, *ETV4*, (3)	*STK36, SUFU*, (1)
9	NF-κB	10	*IKBKE*, *CARD11*, (2)	*IKBKB*, *REL*, et al., (1)	
8	TGF-β	9	*TGFBR2* (3)	*TFE3*, *SMAD4*, *ACVR2A*, (2)	
4	Phosphatases	5	*PTPRT* (4)	*PTPRD* (1)	

The raw sequencing data were processed through a bioinformatics pipeline (GENEPIPER), and the qualified calls/data were uploaded to a knowledgebase (GENEKEEPER) before functional analysis. Each gene with qualified variants from the resulted data set was then subjected to functional mapping and assigned to a signaling pathway or a biological process on the basis of the gene’s primary function. Table 3 shows pathways/biological processes whose genes were mutated in the context of activating EGFR mutations. The associated case number, the total variant number, and the top three mutated genes in each category are shown.

**Table 4:** Mutation Numbers and Types of Top Three Mutated Genes and Their Enrichment in AE cases.

Pathway/Biological Function	Gene	Variant (AA Change)	Variant Number in Total 1565 Cases	Variant Number in 74 AE Cases	Fold Change of Variant Frequency in AE Cases
**Receptors**	*PKHD1*	p.V3934I	3	1	7
		p.T2869K	21	1	1
		p.G2648S	10	1	2
		p.W2280X	1	1	21
		p.T1615M	2	1	10.6
		p.R4036W	5	1	4
		p.R3772Q	1	1	21
		p.S3505R	10	1	2
		p.G448R	7	1	3
		p.G2285E	4	1	5.3
		p.T36M	3	1	7
		p.D220N	1	1	21
		p.E3769D	2	1	10.6
		p.I2364N	35	2	1
		p.R559W	14	1	1.5
		p.V1701I	2	1	10.6
		p.S3210C	35	1	0.6
		p.F2779V	11	1	2
		p.T2082I	8	1	2.6
		p.Q1610K	1	1	21
		**Total**	**176**	**21**	**2.5**
	*ROS1*	p.Q412E	1	1	21
		p.G2121V	1	1	21
		p.K934M	1	1	21
		p.P224S	55	4	1.5
		p.E1902K	44	3	1.4
		p.D618G	4	2	10.6
		p.T326R	9	1	2.3
		p.K461E	1	1	21
		p.L590P	4	1	5.3
		p.A887V	2	1	10
		p.R2039H	5	1	4
		p.D839E	1	1	21
		**Total**	**128**	**18**	**3**
	*RET*	p.T278N	18	2	2.3
		p.L410V	1	1	21
		p.D489N	71	4	1
		p.L56M	16	1	1
		p.T1038A	1	1	21
		**Total**	**107**	**9**	**1.8**
**Genome and Epigenome**	*ARID1A*	p.P21del	53	1	0.4
		p.K1795N	1	1	21
		p.A52V	1	1	21
		p.339_343del	4	1	5.3
		p.L117P	108	1	0.2
		p.Q288P	49	11	4.7
		p.N756fs	1	1	21
		**Total**	**217**	**17**	**1.7**
	*ATM*	p.S49C	19	1	1
		p.T935R	6	1	3.5
		p.I1740V	2	1	10.6
		p.R1730Q	1	1	21
		p.T2640I	2	1	10.6
		p.G1586A	1	1	21
		p.L1450P	1	1	21
		p.S1691R	6	1	3.5
		p.T1756S	1	1	21
		p.K2318Q	2	1	10.6
		p.D1853V	26	1	0.8
		p.I2683fs	1	1	21
		p.G509X	1	1	21
		**Total**	**69**	**13**	**4**
	*BRCA2*	p.A895T	1	1	21
		p.V746G	1	1	21
		p.D1902N	5	1	4
		p.N2436I	10	1	2
		p.K2729N	16	1	1
		p.V2109I	10	1	2
		p.K322Q	21	1	1
		p.M784V	104	1	0.2
		p.G2044V	21	1	1
		p.T3013I	1	1	21
		p.A2351T	2	1	10.6
		**Total**	**192**	**11**	**1.2**
**Wnt/β-catenin**	*FAT1*	p.M1149T	21	1	1
		p.V912I	1	1	21
		p.P509S	1	1	21
		p.S1565F	1	1	21
		p.I2004M	1	1	21
		p.H1850Y	1	1	21
		p.S2353A	13	1	1.6
		p.D1113N	6	1	3.5
		p.T1679I	7	1	3
		p.E1141D	9	1	2.3
		p.I1228V	4	1	5.3
		p.R4208W	4	1	5.3
		**Total**	**69**	**12**	**3.7**
	*APC*	p.V1099I	1	1	21
		p.F1840C	1	1	21
		p.G253S	4	1	5.3
		p.S1545X	1	1	21
		p.K603X	1	1	21
		p.T1585fs	1	1	21
		p.H1845fs	1	1	21
		**Total**	**10**	**7**	**14.8**
	*RNF43*	p.E170K	6	1	3.5
		p.A365T	23	3	2.8
		p.R519Q	15	1	1.4
		p.L214V	1	1	21
		p.R145X	2	1	10.6
		**Total**	**47**	**7**	**3**
	*AXIN2*	p.T673P	34	1	0.6
		p.H474del	13	2	3.3
		p.A761D	5	2	8.5
		p.G755V	1	1	21
		**Total**	**53**	**6**	**2.4**
**TP53/apoptosis**	*TP53*	p.V31I	8	1	2.6
		p.P58fs	1	1	21
		p.S241F	2	1	10.6
		p.V216M	2	1	10.6
		p.R273H	39	1	0.5
		p.Q165X	4	1	5.3
		p.C176F	10	3	6.3
		p.252_254del	2	1	10.6
		p.Q144P	1	1	21
		p.V272M	5	1	4
		p.P72fs	2	1	10.6
		p.P92fs	2	1	10.6
		p.R273P	1	1	21
		p.G154V	7	2	6
		p.R158L	7	1	3
		p.C135Y	3	1	7
		p.P89fs	3	1	7
		p.I232F	2	1	10.6
		p.D281V	2	1	10.6
		p.E258K	1	1	21
		p.V274F	2	1	10.6
		p.Q136X	1	1	21
		p.H179L	3	1	7
		p.K132M	2	1	10.6
		**Total**	**112**	**27**	**5**
	*NUMA1*	p.R972Q	16	1	1.3
		p.L1346M	1	1	21
		p.P1117Q	1	1	21
		p.Y1836H	8	1	2.6
		p.M108I	8	1	2.6
		**Total**	**34**	**5**	**3**
	*NLRP1*	p.P233L	1	1	21
		p.V1231I	12	2	3.5
		**Total**	**13**	**3**	**4.9**
**PI3K–AKT–mTOR**	*PIK3CA*	p.H1048R	1	1	21
		p.I816V	1	1	21
		p.S405F	1	1	21
		p.E545K	44	2	1
		p.E542K	21	1	1
		p.9_18del	1	1	21
		p.G1049R	3	1	7
		p.Y1021C	1	1	21
		p.R108delinsREEKILS	1	1	21
		**Total**	**74**	**10**	**2.9**
	*RICTOR*	p.G1584V	1	1	21
		p.T258fs	1	1	21
		p.D1182G	4	1	5.3
		p.P1668L	13	1	1.6
		p.I518T	1	1	21
		**Total**	**20**	**5**	**5.3**
	*PIK3R2*	p.F381L	1	1	21
		p.P723L	4	1	5.3
		p.P4S	47	1	0.4
		**Total**	**52**	**3**	**1**
	*TSC2*	p.A460T	4	1	5.3
		p.G1787S	2	1	10.6
		p.A678T	8	1	2
		**Total**	**14**	**3**	**4.5**
**NOTCH**	*NOTCH2*	p.P1591L	1	1	21
		p.D182N	1	1	21
		p.R91L	9	1	2.3
		p.Q466K	2	1	10.6
		p.F1209V	12	1	1.8
		p.I1698M	1	1	21
		p.I681N	43	2	1
		**Total**	**69**	**8**	**2.5**
	*NOTCH4*	p.L16delinsLLLL	11	1	2
		p.G487R	1	1	21
		p.1536_1537del	3	1	7
		p.A439P	2	1	10.6
		p.E513D	19	1	1
		p.R1410H	25	3	2.5
		**Total**	**61**	**8**	**2.8**
	*SPEN*	p.D2007E	11	1	2
		p.V1022M	6	1	3.5
		p.N2957D	3	1	7
		p.S286N	1	1	21
		p.R277K	3	1	7
		p.P2240L	36	1	0.6
		p.A2777V	1	1	21
		**Total**	**61**	**7**	**2.4**
	*MAML2*	p.I480M	35	2	1
		p.R422Q	3	2	14
		p.605_607del	13	1	1.6
		p.603_613del	3	1	7
		**Total**	**54**	**6**	**2.3**
**Kinase**	*TNK2*	p.P506R	1	1	21
		p.T829N	4	1	5.3
		p.P584S	4	1	5.3
		p.P887H	2	1	10.6
		p.A701T	4	1	5.3
		p.A104T	5	1	4
		p.R748W	6	1	3.5
		p.R382W	13	1	1.6
		**Total**	**39**	**8**	**4.3**
	*TYK2*	p.E1163G	5	1	4
		p.S418L	1	1	21
		p.R703W	23	4	3.7
		p.R231W	11	1	2
		p.R124H	2	1	10.6
		**Total**	**42**	**8**	**4**
	*ABL1*	p.K247R	2	1	10.6
		p.M237V	1	1	21
		p.K609del	94	2	0.4
		**Total**	**97**	**4**	**0.9**
	*JAK3*	p.R687M	1	1	21
		p.A919S	1	1	21
		p.I688F	2	1	10.6
		p.Q501H	2	1	10.6
		**Total**	**6**	**4**	**14**
	*JAK2*	p.Y1099C	1	1	21
		p.S797C	1	1	21
		p.Q955R	1	1	21
		**Total**	**3**	**3**	**21**
**Rb1/cell cycle**	*RB1*	p.L171fs	1	1	21
		p.F482fs	1	1	21
		p.K192E	1	1	21
		p.A538fs	1	1	21
		**Total**	**4**	**4**	**21**
	*NUP214*	p.S492C	1	1	21
		p.A109V	5	1	4
		p.R741L	12	1	1.8
		p.V1638I	7	1	3
		**Total**	**25**	**4**	**3.4**
	*CDKN2A*	p.R144C	5	1	4
		p.H66R	16	1	1.3
		**Total**	**21**	**2**	**2**
	*CDK12*	p.P1275L	16	2	2.6
		**Total**	**16**	**2**	**2.6**
	*CCND1*	p.276_276del	5	1	4
		**Total**	**5**	**1**	**4**
	*CCND2*	p.R262H	3	1	7
		**Total**	**3**	**1**	**7**
	*CCND3*	p.E253D	25	1	0.8
		**Total**	**25**	**1**	**0.8**
	*AURKA*	p.G173R	1	1	21
		**Total**	**1**	**1**	**21**
	*CDKN1A*	p.P4L	7	1	3
		**Total**	**7**	**1**	**3**
	*CHEK1*	p.V46I	2	1	10.6
		**Total**	**2**	**1**	**10.6**
	*BUB1B*	p.R550Q	8	1	2.6
		**Total**	**8**	**1**	**2.6**
**RAS-RAF-MEK-ERK**	*NF1*	p.Q83L	1	1	21
		p.V1753I	1	1	21
		p.P678T	1	1	21
		p.F2634C	1	1	21
		p.R1958fs	2	1	10.6
		**Total**	**6**	**5**	**17.6**
	*RASA1*	p.E70G	35	4	2.4
		p.G75A	6	1	3.5
		**Total**	**41**	**5**	**2.6**
	*KIAA1804*	p.E563D	11	1	2
		p.I941T	1	1	21
		p.A695V	6	1	3.5
		**Total**	**18**	**3**	**3.5**
	*RPS6KA2*	p.H371Y	5	1	4
		p.R500Q	3	1	7
		**Total**	**8**	**2**	**5.3**
**Hedgehog**	*PTCH1*	p.P1441L	1	1	21
		p.P813A	1	1	21
		p.R893H	16	1	1.3
		p.S649G	1	1	21
		**Total**	**19**	**4**	**4.5**
	*SMO*	p.K575M	12	1	1.8
		p.P698R	6	1	3.5
		p.16_17del	4	1	5.3
		**Total**	**22**	**3**	**2.9**
	*ETV4*	p.H175N	4	2	10.6
		p.V448I	7	1	3
		**Total**	**11**	**3**	**5.8**
	*STK36*	p.H713Y	1	1	21
		**Total**	**1**	**1**	**21**
	*SUFU*	p.R239W	1	1	21
		**Total**	**1**	**1**	**21**
**NF-κB**	*IKBKE*	p.V418M	2	1	10.6
		p.T483M	9	1	2.3
		**Total**	**11**	**2**	**3.8**
	*CARD11*	p.N191S	5	1	4
		p.M551T	1	1	21
		**Total**	**6**	**2**	**7**
	*IKBKB*	p.M83V	2	1	10.6
		**Total**	**2**	**1**	**10.6**
	*REL*	p.L331S	32	1	0.7
		**Total**	**32**	**1**	**0.7**
	*TNFAIP3*	p.C590S	1	1	21
		**Total**	**1**	**1**	**21**
	*BCL11B*	p.D122N	1	1	21
		**Total**	**1**	**1**	**21**
	*BLNK*	p.I308T	2	1	10.6
		**Total**	**2**	**1**	**10.6**
	*BIRC3*	p.R202S	11	1	2
		**Total**	**11**	**1**	**2**
**TGF-β**	*TGFBR2*	p.T315M	12	3	5.3
		**Total**	**12**	**3**	**5.3**
	*TFE3*	p.A139V	2	1	10.6
		p.A484T	4	1	5.3
		**Total**	**6**	**2**	**7**
	*SMAD4*	p.H92Y	2	1	10.6
		p.M447fs	1	1	21
		**Total**	**3**	**2**	**14**
	*ACVR2A*	p.M148T	1	1	21
		p.A151V	1	1	21
		**Total**	**2**	**2**	**21**
**Phosphatases**	*PTPRT*	p.V1017A	9	1	2.3
		p.Q1188fs	1	1	21
		p.S1290F	1	1	21
		p.N1167K	5	1	4
		**Total**	**16**	**4**	**5.3**
	*PTPRD*	p.R1088H1	1	1	21
		**Total**	**1**	**1**	**21**

Table 4 shows the mutation types of top three mutated genes associated with activating mutant EGFRs in each category, and the number of each mutation type detected in 74 AE cases and in total 1565 cases of unselected patient population (in terms of solid tumor types and stages, age, sex, and ethnicity), respectively. The fold change of variant frequency in AE cases is calculated by the mutation frequency in 74 AE cases (# of mutation/74) divided by the mutation frequency in total 1565 cases (# of mutation/1565). More than 2-fold increased total mutation frequency are observed for majority top three mutated genes (88%) in the AE cases, indicating a specific enrichment of these mutations in the AE cases.

**Table 5:** Mutation Distribution of Top Three Mutated Genes between EGFR L858R and *EGFR* exon 19del.

Pathway/Biological Function	Gene	Variant Number in AE Cases	Variant Number in EGFR L858R Cases	Variant Number in *EGFR* Exon 19del Cases	Variant Frequency in L858R Cases (%)	Variant Frequency in Exon 19del Cases (%)
**Receptors**	*PKHD1*	21	6	10	28	47
	*ROS1*	18	3	13	16	72
	*RET*	9	4	3	44	33
**Genome and Epigenome**	*ARID1A*	17	7	7	41	41
	*ATM*	13	6	6	46	46
	*BRCA2*	11	5	4	45	36
**Wnt/β-catenin**	*FAT1*	12	5	5	41	41
	*APC*	7	4	2	57	28
	*RNF43*	7	4	3	57	42
	*AXIN2*	6	0	3	0	50
**TP53/apoptosis**	*TP53*	27	9	13	33	48
	*NUMA1*	5	2	2	40	40
	*NLRP1*	3	0	2	0	66
**PI3K-AKT-mTOR**	*PIK3CA*	10	1	8	10	80
	*RICTOR*	5	2	3	40	60
	*PIK3R2*	3	2	1	66	33
	*TSC2*	3	1	2	33	66
**NOTCH**	*NOTCH2*	8	3	4	37	50
	*NOTCH4*	8	3	4	37	50
	*SPEN*	7	3	2	42	28
	*MAML2*	6	2	3	33	50
**Kinase**	*TNK2*	8	0	5	0	62
	*TYK2*	8	3	4	37	50
	*ABL1*	4	0	3	0	75
	*JAK3*	4	2	1	50	25
	*JAK2*	3	1	1	33	33
**Rb1/cell cycle**	*RB1*	4	1	3	25	75
	*NUP214*	4	1	2	25	50
	*CDKN2A*	2	1	0	50	0
	*CDK12*	2	1	0	50	0
	*CCND1*	1	0	1	0	100
	*CCND2*	1	0	1	0	100
	*CCND3*	1	0	1	0	100
	*AURKA*	1	0	1	0	100
	*CDKN1A*	1	0	1	0	100
	*CHEK1*	1	1	0	100	0
	*BUB1B*	1	1	0	100	0
**RAS-RAF-MEK-ERK**	*NF1*	5	1	2	20	40
	*RASA1*	5	3	2	60	40
	*KIAA1804*	3	2	1	66	33
	*RPS6KA2*	2	0	0	0	0
**Hedgehog**	*PTCH1*	4	0	4	0	100
	*SMO*	3	2	1	66	33
	*ETV4*	3	0	2	0	66
	*STK36*	1	0	1	0	100
	*SUFU*	1	0	1	0	100
**NF-κB**	*IKBKE*	2	2	0	100	0
	*CARD11*	2	1	0	50	0
	*IKBKB*	1	0	1	0	100
	*REL*	1	0	1	0	100
	*TNFAIP3*	1	0	1	0	100
	*BCL11B*	1	1	0	100	0
	*BLNK*	1	1	0	100	0
	*BIRC3*	1	0	1	0	100
**TGF-β**	*TGFBR2*	3	1	1	33	33
	*TFE3*	2	0	2	0	100
	*SMAD4*	2	1	0	50	0
	*ACVR2A*	2	1	1	50	50
**Phosphatases**	*PTPRT*	4	1	3	25	75
	*PTPRD*	1	0	1	0	100

Table 5 shows total variant number of each top three mutated genes in 74 total AE cases, 25 AE cases with EGFR L858R (EGFR L858R cases), and 40 AE cases with *EGFR* exon 19del (*EGFR* exon 19del cases), respectively, as well as fold change of variant frequency in L858R cases [calculated by the mutation frequency in 25 L858R cases (# of mutation/25) divided by the mutation frequency in 74 AE cases (# of mutation/74)], and fold change of variant frequency in exon 19del cases [calculated by the mutation frequency in 40 exon 19del cases (# of mutation/40) divided by the mutation frequency in 74 AE cases (# of mutation/74)].
